# Glutamine – from conditionally essential to totally dispensable?

**DOI:** 10.1186/cc13964

**Published:** 2014-07-02

**Authors:** Jan Wernerman

**Affiliations:** 1 Department of Anesthesia and Intensive Care Medicine, K32, Karolinska University Hospital Huddinge, 14186 Stockholm, Sweden

## Abstract

Recently a large multicentre randomised controlled trial in critically ill patients reported harm to the patients given supplementary glutamine. In the original publication, no explanation was offered for why this result was obtained; a large number of studies have reported beneficial effects or no effect, but never before reported harm. These results have been commented upon in a number of communications. Now some of the authors of the multicentre randomised controlled trial present a review and meta-analysis of glutamine supplementation, and the discrepancy of results is suggested to relate to intravenous administration to patients of supplementary glutamine via parenteral nutrition or a combination of enteral and parenteral nutrition in contrast to enteral administration of supplementation or a combination of enteral and parenteral supplementation. To explain results by epidemiological means only, by combining results into a meta-analysis, is perhaps not the best way to explain mechanisms behind results. Meta-analyses are primarily hypothesis generating. Launching treatment without a solid mechanistic explanation is always risky. Glutamine supplementation of the critically ill comes into that category. Now we will all have to do our homework and try to understand whether supplementation or omission of glutamine for patients fed parenterally is a good idea or not.

## 

The authors of a multicentre randomised controlled trial have recently presented a review and meta-analysis of glutamine supplementation [1]. A discrepancy in results is suggested to relate to intravenous administration to patients of supplementary glutamine via parenteral nutrition or a combination of enteral and parenteral nutrition in contrast to enteral administration of supplementation only.

Supplementation of glutamine to critically ill patients was suggested as level A or level B in most nutrition guidelines [2], but after the Reducing Deaths due to Oxidative Stress (REDOXS) study there is a lot of confusion. In the conclusion to their study, the REDOXS authors state that ‘early administration of glutamine in critically ill patients with multiple organ failure was harmful. The observation that the majority of these patients did not have glutamine deficiency early in the course of their critical illness challenges the prevailing concept that glutamine is an essential nutrient that is deficient in critically ill patients and requires immediate supplementation’ [3]. Several studies have shown that a low glutamine concentration, associated with an unfavourable outcome, is valid for some 30% of ICU patients [4,5]. Comments on the REDOXS study interpretations vary widely – from ‘… should be abandoned in clinical practice’ [6] to ‘… should be reserved for specifically identified patients with compromised glutamine availability’ [7].

There are also comments trying to analyse the REDOXS study results in terms of unbalanced amino acid composition [8] and bias in patient recruitment [9]. Several comments are made after the use of meta-analyses; such as reports that the beneficial effects of glutamine supplementation are confined to single-centre studies [10] or to surgical patients [11]. Recently in *Critical Care* the REDOXS authors themselves published a meta-analysis confined to studies with intravenous glutamine supplementation (thereby excluding their own REDOXS study, which provided glutamine supplementation both by enteral and parenteral routes). The conclusion put forward is that ‘Parenteral glutamine supplementation given in conjunction with nutrition support continues to be associated with a significant reduction in hospital mortality and hospital length of stay. Parenteral glutamine supplementation as a component of nutrition support should continue to be considered to improve outcomes in critically ill patients’ [1].

Obviously different meta-analyses select published studies differently, thereby reaching different conclusions. It is important always to remember the limitations of meta-analyses, and to always remember that they are primarily hypothesis generating – in particular when the treatment is variable in dose, in timing, and in mode of administration, as is the case for supplementary glutamine. There is a giant step from the literature regarding glutamine deficiency in subgroups of critically ill patients as a possible indication for supplementation to outcome studies in unselected groups of critically ill patients. As pointed out, the use of meta-analyses to generate the hypothesis of high-dose glutamine supplementation as a treatment modality was perhaps to rush ahead of solid evidence [12]. The scientific community now has to address the task to explain the REDOXS study results. Is glutamine supplementation harmful, is it a complete waste, or might it be relevant in subgroups of patients?

The merit of the meta-analysis by Wischmeyer and colleagues is that it points out the absence of indications of harm in the studies included, and that potential patient benefits are identified [1]. What is now needed is a thorough search for a possible dose–response relationship in conjunction with glutamine supplementation. Are patients with a low plasma glutamine level at ICU admission candidates for treatment? Should glutamine be given regardless of nutrition support, or as a part of nutrition support? What is proper dosing? The use of meta-analyses to titrate dosing without any measurements of admission glutamine status or post-treatment glutamine status is not good science. Obviously, all of us who have been involved in the glutamine story have to do our homework much better. The first step must be to explore the possible mechanisms by which a glutamine deficiency may be harmful in some patients.

From the REDOXS study itself it is possible to hypothesise from the small subgroup in which glutamine status was actually studied that the toxic effects were not obviously linked to supraphysiologic plasma levels [3]. This hypothesis shifts the focus to the provision of large enteral doses of glutamine, without proper feeding simultaneously. Beneficial effects of large enteral doses together with feeding have been demonstrated by others [13]. One must note, however, that the first-pass elimination of enterally administered glutamine is very high [14,15]. The metabolic fate of supplemented glutamine therefore seems to be different related to the route of administration and related to whether glutamine is given as part of full nutrition or not. These basic metabolic tasks must be addressed before any clinical recommendations are given. To just abandon glutamine without doing this homework properly will not be the best service to our patients. There are too many observations of beneficial effects to just throw glutamine away, and it is just a little too difficult to understand the toxicity of a substance without high plasma levels.

In conclusion, the REDOXS study raises more question than it answers. Studies addressing the underlying mechanisms are needed to determine why glutamine may be toxic without an increase in plasma concentration. Meta-analysis is not the proper method to investigate underlying mechanisms, and also not the proper way to launch clinical recommendations. At best, meta-analysis may serve as hypothesis generating. Following the REDOXS study we all have to do our homework and try to understand the role of glutamine in critical illness, and as a consequence the possible indications and hazards associated with supplementation.

## Abbreviations

REDOXS: Reducing Deaths due to Oxidative Stress.

## Competing interests

JW declares an academic competing interest in being the principal investigator of the Scandinavian Glutamine Trial, during the last 3 years being a recipient of an academic grant to explore the mechanisms behind glutamine supplementation from the Country Council of Stockholm (project 502033), and also in having been a member of a glutamine advisory panel for Fresenius, Bad Homburg, Germany.
